# Therapeutic Modulation of Mitophagy by Cafestol in Pressure Overload-Induced Cardiac Hypertrophy and Fibrosis

**DOI:** 10.3390/nu17233680

**Published:** 2025-11-25

**Authors:** Wen-Rui Hao, Chun-Chao Chen, Guan-Ci Huang, Jia-Hong Lin, Huan-Yuan Chen, Ju-Chi Liu, Tzu-Hurng Cheng, Jin-Jer Chen

**Affiliations:** 1 Division of Cardiology, Department of Internal Medicine, Shuang Ho Hospital, Ministry of Health and Welfare, Taipei Medical University, New Taipei City 23561, Taiwan; b8501043@tmu.edu.tw (W.-R.H.); b101092035@tmu.edu.tw (C.-C.C.); 2 Division of Cardiology, Department of Internal Medicine, School of Medicine, College of Medicine, Taipei Medical University, Taipei 11002, Taiwan; 3 Taipei Heart Institute, Taipei Medical University, Taipei 11002, Taiwan; 4 Division of Gastroenterology, Department of Internal Medicine, Shuang Ho Hospital, Ministry of Health and Welfare, Taipei Medical University, New Taipei City 23561, Taiwan; hjordan100@gmail.com; 5 Taipei Municipal Chenggong High School, Taipei City 100025, Taiwan; shawnlinsogood@gmail.com; 6 Institute of Biomedical Sciences, Academia Sinica, Taipei City 115201, Taiwan; hchen9@ibms.sinica.edu.tw (H.-Y.C.); jc8510@yahoo.com (J.-J.C.); 7 Department of Biochemistry, School of Medicine, College of Medicine, China Medical University, Taichung 404328, Taiwan; 8 Division of Cardiology, Department of Internal Medicine and Graduate Institute of Clinical Medical Science, China Medical University, Taichung City 404328, Taiwan

**Keywords:** cafestol, mitophagy, pressure overload, fibrosis

## Abstract

**Background/Objectives**: Mitophagy, the selective removal of damaged mitochondria, plays a pivotal role in regulating cardiac hypertrophy and fibrosis under pressure overload. Targeting mitophagy may help mitigate adverse cardiac remodeling. This preclinical study examined the effects of cafestol, a coffee-derived diterpene, on pressure overload-induced cardiac hypertrophy and fibrosis in mice, with emphasis on mitophagy modulation and mitochondrial ultrastructure. **Methods**: Male normotensive mice underwent transverse aortic constriction (TAC) and received cafestol at 2, 10, or 50 mg/kg/day via oral gavage for 28 days. Cardiac function was assessed by echocardiography. Histological and molecular analyses quantified fibrosis, inflammation, and apoptosis. Protein expression of CD68, CTGF, DDR2, α-SMA, CD44, galectin-3 (Gal3), collagen I, GAPDH, Bcl-2, Bax, cleaved caspase-3, GRP78, p-ERK/ERK, ATF4, p-mTOR/mTOR, and p62 was evaluated. Transmission electron microscopy (TEM) was used to assess autophagosome formation and mitochondrial morphology. **Results**: TAC induced significant cardiac hypertrophy and fibrosis, accompanied by elevated expression of fibrotic (CTGF, DDR2, α-SMA, collagen I), inflammatory (CD68, CD44, Gal3), apoptotic (Bax, cleaved caspase-3), and endoplasmic reticulum stress markers (GRP78, ATF4). TEM revealed increased autophagosome accumulation and disrupted mitochondrial architecture. Cafestol treatment reduced collagen deposition, immune cell infiltration, and apoptotic signaling; enhanced Bcl-2 expression; and restored p62 levels. TEM findings demonstrated decreased autophagosome burden and preserved mitochondrial structure, consistent with improved mitophagic flux and mitochondrial homeostasis. **Conclusions**: Cafestol mitigated pressure overload-induced cardiac remodeling in mice by modulating mitophagy, suppressing fibrotic and inflammatory responses, and preserving mitochondrial integrity. These findings support further investigation of cafestol’s mechanisms and safety profile in preclinical models of cardiovascular disease.

## 1. Introduction

Cardiac remodeling is a dynamic biological process that plays a central role in the onset and progression of heart failure (HF). Initiated by chronic hemodynamic stress, particularly systemic hypertension, the myocardium undergoes structural and functional modifications designed to maintain adequate cardiac output. Early adaptations, such as cardiomyocyte hypertrophy and extracellular matrix expansion, act as compensatory mechanisms to stabilize ventricular performance [1]. However, sustained pressure overload eventually transforms these adaptive responses into maladaptive remodeling, characterized by increased myocardial stiffness, impaired contractility, and progressive ventricular dysfunction, ultimately culminating in HF [2,3].

Recent studies have identified cafestol, a diterpene compound naturally present in unfiltered coffee, as a bioactive molecule with diverse pharmacological properties relevant to cardiovascular health. Beyond its well-documented antioxidant and anti-inflammatory activities, cafestol has demonstrated organ-protective effects in ischemia–reperfusion models, where it mitigated renal injury by modulating oxidative stress and inflammatory signaling [4]. Its proven bioaccessibility and metabolic stability in in vitro digestion models further support its potential for systemic therapeutic use [5]. Cardiovascular studies have shown that cafestol activates the Nrf2 pathway, a central regulator of cellular defense mechanisms, thereby attenuating oxidative injury and fibrotic progression [6,7]. Liu et al. reported that cafestol suppressed high-glucose-induced cardiac fibrosis in diabetic rats, underscoring its relevance in both metabolic and pressure-overload conditions [8]. In addition, cafestol’s ability to inhibit proinflammatory cytokine production and endothelial adhesion molecule expression under mechanical strain highlights its anti-inflammatory potential in vascular remodeling [9].

Mitophagy, the selective autophagic degradation of damaged mitochondria, plays a pivotal role in maintaining cardiac homeostasis under stress conditions. Under pressure overload, impaired mitophagy contributes to mitochondrial dysfunction, oxidative stress, and maladaptive remodeling, thereby exacerbating cardiac hypertrophy and fibrosis. Numerous studies have highlighted the therapeutic relevance of mitophagy modulation in cardiovascular disease models. For instance, semaglutide alleviated pressure overload-induced cardiac hypertrophy by enhancing mitophagy and suppressing NLRP3 inflammasome activation, thereby reducing inflammation and fibrotic progression [10]. Similarly, α-ketoglutarate improved cardiac function in pressure-overloaded mice through NAD^+^–SIRT1 signaling, promoting mitophagy and mitigating ferroptosis [11]. Natural compounds and pharmacological agents have also demonstrated efficacy in restoring mitochondrial quality. Berberine enhanced cardiac performance by upregulating PINK1/Parkin-mediated mitophagy in heart failure models [12], while alpha-lipoic acid conferred cardioprotection via ALDH2-dependent Nrf1–FUNDC1 signaling [13]. Fenofibrate preserved mitochondrial dynamics and attenuated cardiac remodeling in renovascular hypertrophy, underscoring the therapeutic value of maintaining mitochondrial integrity [14]. Traditional formulations such as Xinyang tablet—a compound preparation containing *Salvia miltiorrhiza*, *Panax notoginseng*, and *Ligusticum chuanxiong*—have shown promise in modulating the mitochondrial unfolded protein response and FUNDC1-dependent mitophagy, thereby improving cardiac performance under pressure overload [15]. Likewise, metformin, acting synergistically with PINK1/Mfn2 overexpression, prevented cardiac injury by enhancing mitochondrial function and autophagic flux [16]. Dietary factors also influence mitophagy: short-term high-fat diet intake activated mitophagy and protected against pressure overload-induced heart failure, whereas long-term intake reversed these benefits [17]. Resveratrol further promoted mitophagy and inhibited apoptosis in uremic toxin-induced intestinal barrier dysfunction, suggesting broader systemic implications for mitochondrial quality control [18]. Emerging evidence also implicates cafestol in autophagy regulation. Feng et al. demonstrated that cafestol activated LKB1/AMPK/ULK1-dependent autophagy in colon cancer models, a pathway closely linked to mitophagy and mitochondrial quality control [19]. Given the central role of mitophagy in sustaining cardiac homeostasis under stress, these findings suggest that cafestol exerts cardioprotective effects by promoting mitochondrial turnover and limiting maladaptive remodeling. Collectively, the evidence supports the hypothesis that cafestol functions as a multifaceted modulator of cardiac remodeling through antioxidative, anti-inflammatory, and mitophagy-related mechanisms. Its favorable safety profile and metabolic accessibility further strengthen its candidacy for translational research in cardiovascular therapeutics [20].

The aim of this study was to clarify the therapeutic role of cafestol in pressure overload-induced cardiac pathology by evaluating its effects on structural remodeling, inflammatory responses, and mitochondrial regulation. Through integrated histological, biochemical, and molecular analyses, we provide deeper insight into how cafestol counteracts the progression of heart failure by coordinating the modulation of fibrosis, inflammation, and mitophagy. These findings support further investigation of cafestol as a promising candidate for translational research in cardiovascular therapeutics.

## 2. Materials and Methods

### 2.1. Materials

Cafestol was obtained from Sigma-Aldrich (St. Louis, MO, USA). A broad panel of primary antibodies was employed to detect proteins involved in inflammation, fibrosis, apoptosis, and cellular stress responses. These included galectin-3 (Gal3; R&D Systems, Minneapolis, MN, USA; Cat# MAB1197), a marker associated with fibrotic and inflammatory signaling in cardiac tissue; CD68 (Bioss, Woburn, MA, USA; Cat# bs-0649R), a macrophage marker; connective tissue growth factor (CTGF; Santa Cruz Biotechnology, Dallas, TX, USA; Cat# sc-14939); discoidin domain receptor 2 (DDR2; Santa Cruz Biotechnology, Dallas, TX, USA; Cat# sc-8989); alpha-smooth muscle actin (α-SMA; Abcam, Cambridge, UK; Cat# ab5694); and CD44 (MedChemExpress [MCE], Monmouth Junction, NJ, USA; Cat# HY-P80062), which mediates cell adhesion and fibrotic remodeling. Additional targets included collagen I (Santa Cruz Biotechnology, Dallas, TX, USA; Cat# sc-59772), a key extracellular matrix component; GAPDH (Cell Signaling Technology, Danvers, MA, USA; Cat# 2118), used as a loading control; and apoptosis-related proteins Bcl-2, Bax, and cleaved caspase-3. Stress and autophagy markers such as GRP78, ATF4, p62, and phosphorylated/total ERK and mTOR were also assessed. Secondary detection was performed using horseradish peroxidase (HRP)-conjugated anti-mouse IgG (BioLegend, San Diego, CA, USA; Cat# B426166), optimized for chemiluminescent visualization. Protein concentrations were measured using the Bicinchoninic Acid (BCA) Protein Assay Kit (Thermo Scientific, Waltham, MA, USA; Cat# 23228). Western blot signals were developed using Immobilon Western Chemiluminescent HRP Substrate (Millipore, Billerica, MA, USA). Histological evaluation included Sirius Red staining (Sigma-Aldrich, St. Louis, MO, USA; Cat# 365548) for collagen detection and Masson’s Trichrome staining (Sigma-Aldrich, St. Louis, MO, USA; Cat# 41116121) to assess fibrotic architecture.

### 2.2. Animals and Experimental Model

All animal procedures were conducted in accordance with the U.S. National Institutes of Health Guidelines for the Care and Use of Laboratory Animals (NIH Publication, revised 2011) and were approved by the Institutional Animal Care and Use Committee (IACUC) of Taipei Medical University (IACUC 2022-0388; approval date: 2 May 2023). A total of 25 male *Mus musculus* (C57BL/6J strain; 8–10 weeks old, 23.5–27.5 g) were obtained from the Institute of Laboratory Animal Science, Academia Sinica, Taipei, Taiwan. All animals were specific pathogen-free (SPF) and housed in an AAALAC-accredited facility under controlled temperature, humidity, and light–dark cycles. Mice were randomly assigned to five experimental groups (*n* = 5 per group) using a computer-generated randomization sequence. Blinding was implemented throughout the study: both group allocation and outcome assessments were performed by personnel blinded to treatment conditions. To model pressure overload-induced cardiac hypertrophy and heart failure, mice underwent transverse aortic constriction (TAC) or sham surgery (Sham) following established protocols that reliably reproduce these pathophysiological features in vivo [21]. Anesthesia was induced via intraperitoneal injection of pentobarbital sodium (80 mg/kg, 3% solution; Sigma-Aldrich, Cat# T48402), ensuring adequate depth for thoracic intervention. Following intubation, animals were mechanically ventilated at 105 breaths per minute with airway pressure maintained between 13–15 cm H_2_O to stabilize respiratory function. The surgical field was sterilized, and a 5 mm incision was made at the left second or third intercostal space. Soft tissue and muscle layers were carefully separated to expose the thoracic cavity. The intercostal muscle was dissected approximately 2 mm lateral to the sternum, and the chest was gently expanded using a retractor to visualize the descending thoracic aorta. A 6-0 silk suture was looped around the aorta and tied securely to induce constriction, thereby simulating chronic pressure overload. The needle was withdrawn, and the thoracic cavity was closed in anatomical layers. Postoperative care included thermal support and continuous monitoring until recovery. Sham-operated mice underwent identical procedures except for the ligation step, serving as baseline controls for comparative analysis. Cafestol was dissolved in 0.5% carboxymethylcellulose and administered via oral gavage once daily for 28 consecutive days following TAC surgery. The doses used—2, 10, and 50 mg/kg/day (designated as Cafestol-L, Cafestol-M, and Cafestol-H)—were selected based on prior studies demonstrating bioactivity and tolerability in rodent models of inflammation and fibrosis [8]. Blinding was implemented throughout the study: group allocation and outcome assessments were performed by personnel blinded to treatment conditions. At the end of the experimental period, mice were euthanized via intraperitoneal overdose of pentobarbital sodium (150 mg/kg), followed by thoracotomy to ensure complete euthanasia in accordance with IACUC guidelines.

### 2.3. Echocardiographic and Hemodynamic Assessment

To evaluate cardiac morphology and function following sham or TAC surgery, transthoracic echocardiography was performed using a Philips IE-33 imaging system equipped with a 25 MHz RMV-710 transducer [21]. M-mode recordings at the mid-papillary level were obtained to quantify key parameters, including left ventricular end-diastolic diameter (LVEDd), end-systolic diameter (LVESd), and ejection fraction (LVEF), thereby enabling precise assessment of ventricular dimensions and systolic performance under pressure overload conditions. Echocardiographic measurements were analyzed by blinded investigators to minimize bias. To complement these imaging data, invasive hemodynamic measurements were acquired via in vivo left ventricular catheterization. Mice were anesthetized with 1.5% isoflurane to maintain physiological stability during data collection. A microtip pressure transducer catheter was advanced through the right carotid artery into the left ventricular chamber, allowing continuous recording of intraventricular pressure and heart rate. Hemodynamic data were processed using LabChart 7 software (ADInstruments, Dunedin, New Zealand), providing direct insight into cardiac contractility and chamber pressure. This dual-modality approach, combining noninvasive echocardiographic imaging with invasive pressure monitoring, offers a rigorous framework for characterizing structural and functional cardiac changes in murine models of pressure overload. The methodology aligns with established criteria for investigating ventricular remodeling and inflammatory responses in vivo, thereby ensuring reproducibility and transparency.

### 2.4. Histological Evaluation

Cardiac tissues were processed using a standardized histological workflow to evaluate structural alterations and fibrotic remodeling [21]. After fixation, samples were dehydrated through a graded ethanol series and embedded in paraffin to preserve tissue architecture. Thin sections (~5 μm) were prepared and stained with Sirius Red to visualize general histological features and with Masson’s Trichrome to delineate collagen fibers, a key indicator of myocardial fibrosis. Collagen content was quantified by calculating the percentage of Sirius Red- or Masson’s Trichrome-stained area relative to the total myocardial area using Image Pro Plus software (version 6.0; Media Cybernetics, Bethesda, MD, USA), following established fibrosis assessment protocols. Inflammatory infiltration was assessed by immunohistochemical staining for CD68, a macrophage marker, and quantified by measuring the area occupied by CD68-positive immune cells relative to the total tissue area. Stained sections were imaged using a Leica DM4000B light microscope (Leica Microsystems, Wetzlar, Germany), ensuring high-resolution visualization of histopathological features. Quantitative analysis was performed using Image Pro Plus software (version 6.0; Media Cybernetics, Bethesda, MD, USA), enabling precise measurement of fibrotic and inflammatory regions. This dual-staining approach, combining Sirius Red and Masson’s Trichrome, provides a robust framework for evaluating myocardial injury and fibrosis. It also supports interpretation of therapeutic effects targeting inflammation and extracellular matrix remodeling in cardiovascular disease models, thereby enhancing reproducibility and transparency.

### 2.5. Western Blot Analysis

Cardiac tissues were harvested and immediately rinsed with ice-cold phosphate-buffered saline to preserve protein integrity. Samples were lysed using radioimmunoprecipitation assay (RIPA) buffer supplemented with protease and phosphatase inhibitors, including phenylmethylsulfonyl fluoride, to prevent enzymatic degradation and maintain post-translational modifications relevant to inflammatory and stress signaling pathways. Protein concentrations were determined using the BCA Protein Assay Kit (Thermo Scientific), ensuring accurate quantification for consistent gel loading. Equal amounts of protein were separated on 10% SDS-polyacrylamide gels and transferred to polyvinylidene fluoride (PVDF) membranes, which provide high binding efficiency for immunodetection. Membranes were blocked with 5% non-fat dry milk in Tris-buffered saline containing 0.1% Tween-20 (TBST) for 90 minutes to reduce nonspecific binding. Primary antibodies were diluted in TBST and incubated overnight at 4 °C. The antibody panel included markers of inflammation (CD68, Gal3), fibrosis (CTGF, DDR2, α-SMA, CD44, collagen I), apoptosis (Bcl-2, Bax, cleaved caspase-3), and cellular stress and autophagy (GRP78, ATF4, p62, phosphorylated and total ERK, phosphorylated and total mTOR). GAPDH was used as a housekeeping protein for normalization. After primary incubation, membranes were washed and incubated with HRP-conjugated secondary antibodies (1:20,000 dilution) for one hour. Protein bands were visualized using enhanced chemiluminescence substrate and imaged with the Fusion FX5 Spectra system (Vilber Lourmat, Collégien, France), enabling sensitive detection of low-abundance targets.

### 2.6. Transmission Electron Microscopy (TEM)

To evaluate mitochondrial ultrastructure and mitophagic activity in the context of cardiac hypertrophy, TEM was performed using established protocols optimized for subcellular imaging in pressure overload models. Left ventricular tissue samples were promptly harvested and fixed in 2.5% glutaraldehyde buffered with 0.1 M sodium cacodylate (pH 7.4) at 4 °C for 24 hours to preserve organelle integrity. Post-fixation was carried out with 1% osmium tetroxide for 1 hour, followed by sequential dehydration in graded ethanol and embedding in epoxy resin. Ultrathin sections (~70 nm) were prepared using an ultramicrotome and mounted on copper grids. Contrast enhancement was achieved by staining with uranyl acetate and lead citrate. Imaging was conducted using a FEI Tecnai G2 F20 S-TWIN transmission electron microscope (FEI Company, Hillsboro, OR, USA), with magnifications ranging from 5000× to 30,000× to visualize mitochondrial morphology, autophagic structures, and cristae organization. Key indicators of mitophagy—including double-membrane autophagosomes surrounding mitochondria, swelling, cristae disruption, and vacuolar degeneration—were assessed according to criteria established in prior studies of cardiac remodeling. Quantitative analysis was performed using ImageJ software (version 1.53; National Institutes of Health, Bethesda, MD, USA). To ensure reproducibility and minimize bias, blinded observers independently evaluated autophagosome frequency and the proportion of mitochondria exhibiting structural compromise. This methodology provided high-resolution characterization of mitochondrial dynamics and offered mechanistic insight into cafestol’s role in preserving organelle quality under hypertrophic stress.

### 2.7. Statistical Analysis

All quantitative data were analyzed using SPSS software version 22.0 (SPSS Inc., Chicago, IL, USA) and GraphPad Prism version 8.0 (GraphPad Software, San Diego, CA, USA). Results are expressed as mean ± standard deviation (mean ± SD). Prior to statistical testing, data distributions were assessed for normality using the Shapiro–Wilk test, and homogeneity of variance was evaluated with Levene’s test. Group differences were analyzed by one-way analysis of variance (ANOVA), followed by Tukey’s honestly significant difference (HSD) test or Bonferroni correction for multiple comparisons, as appropriate. Effect sizes (*η*^2^) and 95% confidence intervals (CI) were calculated to aid interpretation of statistical outcomes. A significance threshold of *p* < 0.05 was applied throughout the study. All experiments were conducted with randomization and blinding to minimize bias. For in vivo experiments, sample sizes were *n* = 5 per group, determined based on estab-lished protocols for murine TAC models and optimized to balance assay reproducibil-ity with statistical power. For Western blot analyses, sample sizes were *n* = 3 per group, consistent with standard practice for molecular assays. These measures ensured transparency, reproducibility, and methodological rigor in the analysis of experimental outcomes.

## 3. Results

### 3.1. Cafestol Attenuates Cardiac and Ventricular Enlargement After Transverse Aortic Constriction-Induced Hypertrophy

**Figure 1:**
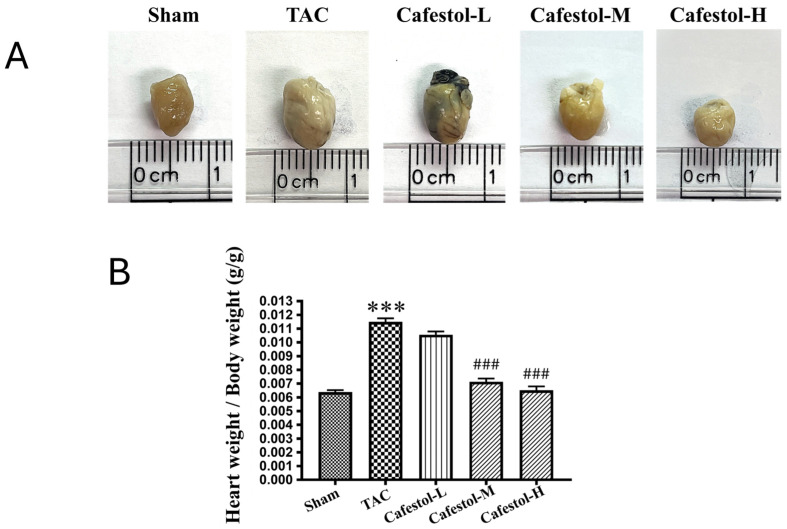
Effects of cafestol on cardiac morphology and weight parameters under different experimental conditions. (**A**) Representative images of excised hearts from each group illustrate the morphological impact of cafestol treatment. (**B**) Quantitative analysis of heart weight-to-body weight ratios shows significant increases following TAC, consistent with hypertrophic remodeling. Cafestol administration at 2 mg/kg/day (Cafestol-L), 10 mg/kg/day (Cafestol-M), and 50 mg/kg/day (Cafestol-H) attenuated these elevations compared with the untreated TAC group. Experimental groups included sham-operated controls, TAC-only mice, and TAC mice treated with cafestol at the indicated doses. Data are presented as mean ± SD (*n* = 5 per group). Statistical comparisons were performed using one-way ANOVA followed by Tukey’s post hoc test. Normality and variance homogeneity were confirmed using the Shapiro–Wilk and Levene’s tests. Effect sizes (*η*^2^) and 95% confidence intervals (CI) were calculated to support interpretation. *** *p* < 0.001 vs. sham; ### *p* < 0.001 vs. TAC.

Cafestol exerted a significant effect on TAC-induced cardiac hypertrophy. Representative heart images revealed clear morphological differences among experimental groups, with a visible reduction in heart and left ventricular size following cafestol treatment (Figure 1). Quantitative analysis confirmed that TAC surgery markedly increased heart and left ventricular weights; however, these increases were significantly attenuated in cafestol-treated mice, indicating a reversal of pressure overload-induced hypertrophic remodeling. The observed cardioprotective effects are likely attributable to cafestol’s ability to modulate oxidative stress and inflammatory signaling, thereby limiting pathological myocardial enlargement.

### 3.2. Cafestol Improves Cardiac Performance in TAC-Induced Hypertrophy

**Figure 2:**
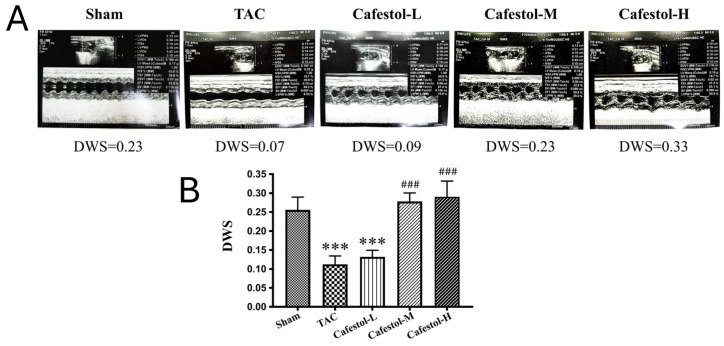
Impact of cafestol on cardiac function in a murine model of TAC-induced hypertrophy. (**A**) Representative echocardiographic images from each group illustrate alterations in cardiac performance following TAC and cafestol treatment. (**B**) Quantitative assessment of diastolic wall strain (DWS) revealed significant group differences. TAC surgery markedly reduced left ventricular ejection fraction compared with sham-operated controls, whereas cafestol administration at 2 mg/kg/day (Cafestol-L), 10 mg/kg/day (Cafestol-M), and 50 mg/kg/day (Cafestol-H) partially restored systolic function. DWS was calculated as [PWT(systole) − PWT(diastole)]/PWT(systole), serving as an index of left ventricular diastolic stiffness. Data are presented as mean ± SD (*n* = 5 per group). Statistical comparisons were performed using one-way ANOVA followed by Tukey’s post hoc test. Normality and variance assumptions were verified with the Shapiro–Wilk and Levene’s tests. Effect sizes (*η*^2^) and 95% confidence intervals (CI) were calculated to support interpretation. *** *p* < 0.001 vs. sham; ### *p* < 0.001 vs. TAC.

Cardiac function was assessed by echocardiographic imaging and ventricular morphometry in mice subjected to TAC. Cafestol administration visibly improved cardiac structure and function across treatment groups (Figure 2). Notably, diastolic wall strain was significantly increased in cafestol-treated mice, and left ventricular ejection fraction partially recovered compared with untreated TAC mice. These functional improvements suggest that cafestol protects against pressure overload-induced dysfunction. Quantitative data (Table 1) supported these observations: TAC-induced increases in left ventricular end-diastolic and end-systolic volumes were markedly reduced following cafestol treatment, demonstrating its capacity to limit pathological ventricular dilation. In addition, cafestol moderated changes in left ventricular internal dimensions at systole and diastole as well as posterior wall thickness, indicating a stabilizing effect on myocardial remodeling. Measurements of interventricular septal thickness at end-diastole and end-systole remained relatively stable across all groups (Table 1), suggesting that the structural effects of cafestol may be regionally selective.

### 3.3. Cafestol Mitigates Histopathological Alterations in TAC-Induced Cardiac Hypertrophy

**Figure 3:**
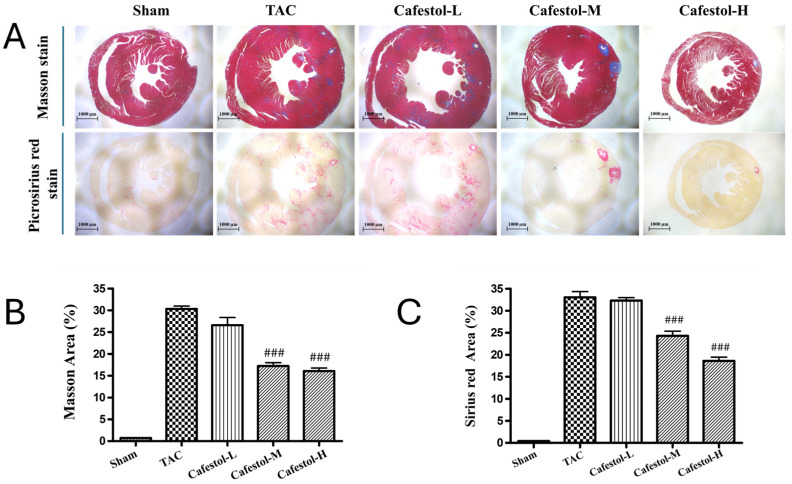
Histological evaluation of cafestol’s impact on myocardial integrity and fibrosis in TAC-induced cardiac hypertrophy. (**A**) Representative micrographs of left ventricular sections stained with Masson’s trichrome (top panels) and Sirius Red (bottom panels), captured at 200× magnification. Scale bars = 100 μm. (**B**,**C**) Quantitative analysis of myocardial injury and collagen deposition is presented as percentage area measurements. Data are shown as mean ± SD (*n* = 5 per group). Statistical comparisons were performed using one-way ANOVA with Tukey’s post hoc test. ### *p* < 0.01 vs. TAC group.

Histological examination of myocardial tissue revealed pronounced structural disruption in mice subjected to TAC. Masson’s trichrome and Sirius Red staining demonstrated extensive inflammatory infiltration, necrotic foci, and disorganized myocardial architecture in the TAC group, accompanied by substantial collagen accumulation indicative of fibrotic remodeling (Figure 3). Cafestol treatment markedly alleviated these pathological changes. Collagen deposition in the left ventricle was significantly reduced following cafestol administration, as reflected by a lower percentage of fibrotic areas, suggesting a protective effect against fibrosis. The reduction in collagen accumulation and preservation of myocardial architecture highlight the potential of cafestol to mitigate fibrotic progression in pressure overload-induced hypertrophy.

### 3.4. Cafestol Regulates Fibrotic and Inflammatory Signaling in TAC-Induced Cardiac Remodeling

**Figure 4:**
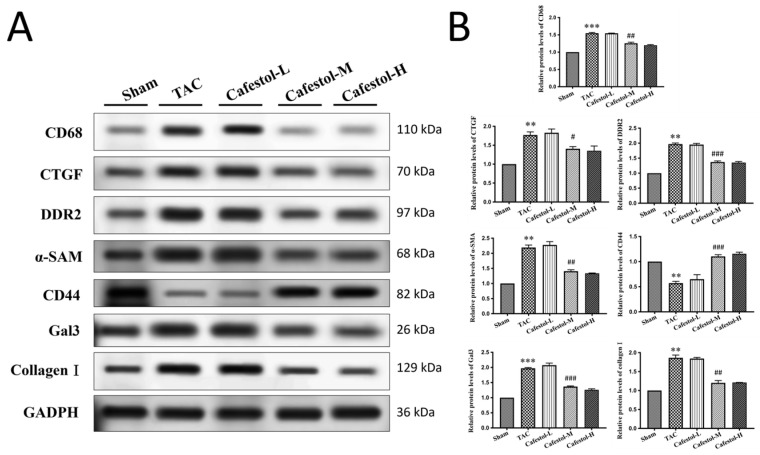
Cafestol’s effects on fibrotic and inflammatory protein expression in TAC-induced cardiac hypertrophy. (**A**) Representative Western blot images showing expression levels of Gal3, CD68, CTGF, DDR2, α-SMA, collagen I, and CD44 across experimental groups. GAPDH served as a loading control. (**B**) Quantitative bar charts summarize densitometric analysis of protein bands. TAC surgery elevated fibrotic and inflammatory markers, whereas cafestol treatment reduced their expression and restored CD44 levels. Data are presented as mean ± SD (*n* = 3 per group). Statistical analysis was performed using one-way ANOVA with Tukey’s post hoc test. Normality and variance assumptions were verified using the Shapiro–Wilk and Levene’s tests. ** *p* < 0.01, *** *p* < 0.001 vs. sham controls; # *p* < 0.05, ## *p* < 0.01, ### *p* < 0.001 vs. TAC group.

**Figure 5:**
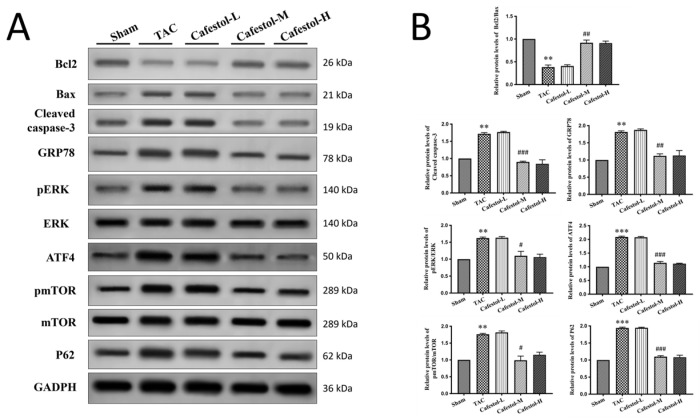
Cafestol’s regulatory effects on apoptosis, endoplasmic reticulum (ER) stress, and signaling pathways in TAC-induced cardiac hypertrophy. (**A**) Representative Western blot images showing expression levels of Bcl-2, Bax, cleaved caspase-3, GRP78, ATF4, p62, phosphorylated and total ERK (p-ERK, ERK), and phosphorylated and total mTOR (p-mTOR, mTOR) across experimental groups. GAPDH served as a loading control. (**B**) Bar charts present densitometric quantification of protein expression. TAC surgery elevated pro-apoptotic and stress markers while suppressing protective signaling components, whereas cafestol treatment reversed these changes, indicating its role in modulating cell survival and stress adaptation pathways. Data are presented as mean ± SD (*n* = 3 per group). Statistical analysis was performed using one-way ANOVA with Tukey’s post hoc test. Normality and variance assumptions were verified using the Shapiro–Wilk and Levene’s tests. ** *p* < 0.01, *** *p* < 0.001 vs. sham controls; # *p* < 0.05, ## *p* < 0.01, ### *p* < 0.001 vs. TAC group.

Western blot analysis revealed pronounced upregulation of fibrosis- and inflammation-related markers in response to TAC. TAC surgery significantly increased the expression of Gal3, CD68, CTGF, DDR2, α-SMA, and collagen I—hallmarks of pro-fibrotic and inflammatory activation (Figure 4; see Supplementary Figure S1 for crude immunoblotting data supporting protein expression). Cafestol treatment markedly suppressed the expression of these proteins, indicating its ability to mitigate maladaptive remodeling processes. Notably, cafestol restored CD44 expression, which was diminished in TAC-induced hypertrophy. Given the role of CD44 in cell adhesion and migration during tissue repair, this restoration suggests a specific mechanism through which cafestol facilitates structural recovery and limits fibrotic progression. Broader pathway analysis (Figure 5) demonstrated that cafestol modulated key regulators of cellular stress and apoptosis. Cafestol treatment reduced the expression of proapoptotic markers such as Bax and cleaved caspase-3, while maintaining the expression of the antiapoptotic marker Bcl-2. In addition, cafestol influenced endoplasmic reticulum stress and autophagy-related proteins, including GRP78, ATF4, and p62, as well as components of the mTOR and ERK signaling cascades (p-mTOR, mTOR, p-ERK, and ERK). These findings suggest that cafestol exerts a multifaceted role in maintaining mitochondrial and cellular homeostasis. Collectively, the results highlight the therapeutic potential of cafestol in pressure overload-induced cardiac hypertrophy. By modulating inflammatory, fibrotic, apoptotic, and stress-responsive pathways, cafestol appears to interrupt the progression of pathological remodeling and enhance myocardial resilience.

### 3.5. Transmission Electron Microscopy Reveals Cafestol-Mediated Mitochondrial Protection in Hypertrophic Hearts

**Figure 6:**
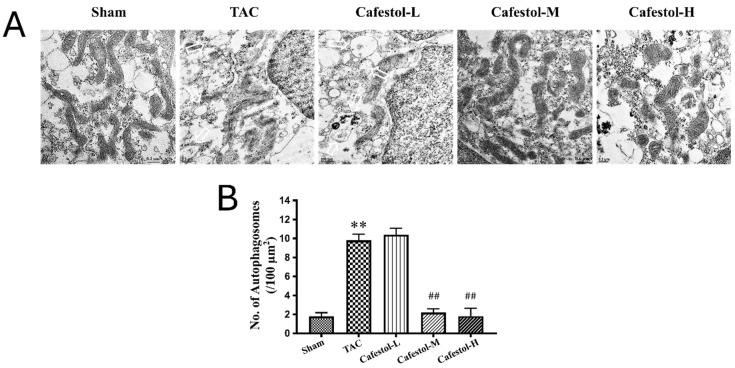
Ultrastructural assessment of mitophagy and mitochondrial integrity in left ventricular myocardium following TAC and cafestol treatment. (**A**) TEM images from sham-operated (Sham), TAC, and cafestol-treated mice (2, 10, and 50 mg/kg/day; Cafestol-L, Cafestol-M, and Cafestol-H) illustrate distinct morphological changes. TAC hearts exhibited swollen mitochondria, disrupted cristae, and increased autophagosome formation, indicative of organelle stress and impaired mitophagic activity. White arrows denote autophagosomes. Cafestol administration preserved mitochondrial architecture and reduced autophagosome density, suggesting enhanced mitophagic flux and structural stabilization. (**B**) Quantitative analysis of autophagosome density and mitochondrial integrity across experimental groups. Data are presented as mean ± SD (*n* = 5 per group). Statistical comparisons were performed using one-way ANOVA with Tukey’s post hoc test. ** *p* < 0.01 vs. sham controls; ## *p* < 0.01 vs. TAC group.

TEM provided ultrastructural insights into mitochondrial remodeling in response to TAC. Myocardial tissue from TAC-operated mice exhibited pronounced mitochondrial swelling, disrupted cristae organization, and autophagosome accumulation—features indicative of impaired mitophagic clearance and heightened organelle stress (Figure 6A). These alterations reflect the substantial cellular burden imposed by pressure overload. Cafestol treatment at 10 and 50 mg/kg/day markedly improved mitochondrial morphology. TEM images revealed preserved cristae, reduced mitochondrial swelling, and fewer autophagosomes in cafestol-treated hearts, suggesting enhanced mitophagic flux and improved organelle quality control. Quantitative analysis of autophagosome density and mitochondrial integrity (Figure 6B) corroborated these observations. Compared with the TAC control group, cafestol-treated mice exhibited significantly lower autophagosome counts and improved mitochondrial preservation. Collectively, these findings indicate that cafestol promotes mitochondrial resilience and mitigates ultrastructural damage associated with cardiac hypertrophy.

## 4. Discussion

This study demonstrated that cafestol confers robust mitochondrial quality-associated cardioprotection in a murine model of pressure overload-induced cardiac remodeling. TAC provoked pronounced cardiac hypertrophy, interstitial fibrosis, inflammatory infiltration, and apoptotic activation—hallmarks of maladaptive structural and molecular remodeling. Cafestol administration significantly mitigated these pathological changes, as evidenced by reduced collagen deposition, downregulation of fibrotic markers (CTGF, DDR2, α-SMA, and collagen I), and suppression of inflammatory mediators (CD68, CD44, and Gal3). Furthermore, cafestol attenuated apoptosis by decreasing Bax and cleaved caspase-3 expression while increasing Bcl-2 levels, indicating a shift toward prosurvival signaling. Restoration of mitophagic flux and mitochondrial integrity emerged as central mechanisms underlying cafestol’s therapeutic efficacy. TAC-induced autophagosome accumulation and mitochondrial disruption were markedly reversed following cafestol treatment. The normalization of p62 expression, coupled with preserved mitochondrial morphology, supports enhanced autophagic clearance and organelle homeostasis. Together, these findings suggest that cafestol alleviates pressure overload-induced cardiac dysfunction by suppressing fibrotic, inflammatory, and apoptotic pathways while promoting mitophagy and mitochondrial resilience.

### 4.1. Mitophagy as a Therapeutic Target

**Figure 7:**
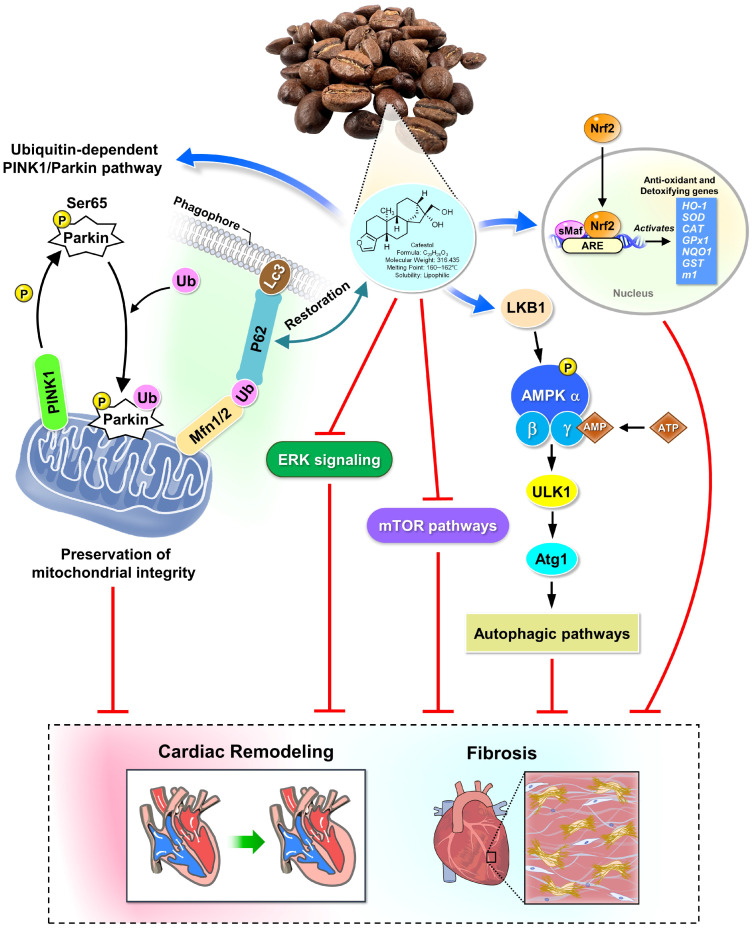
Schematic overview of cafestol’s mechanistic actions. This schematic illustrates the integrated molecular pathways through which cafestol safeguards cardiac function. Cafestol activates the liver kinase B1 (LKB1)–AMP-activated protein kinase (AMPK) –Unc-51 like autophagy activating kinase 1 (ULK1; mammalian homolog of autophagy-related protein 1 [Atg1]) axis and promotes PTEN-induced kinase 1 (PINK1)–Parkin–sequestosome 1 (p62)–mediated mitophagy, facilitating mitochondrial turnover and alleviating organelle stress. In parallel, cafestol enhances nuclear factor erythroid 2–related factor 2 (Nrf2)–driven antioxidant defenses. Upon oxidative stress, Nrf2 translocates into the nucleus and binds to the antioxidant response element (ARE), initiating transcription of antioxidant and cytoprotective genes including heme oxygenase-1 (HO-1), superoxide gismutase (SOD), catalase (CAT), glutathione peroxidase 1 (GPx1), NAD(P)H quinone dehydrogenase 1 (NQO1), glutathione S-transferase (GST), and Maf1 homolog (m1). Concurrently, cafestol suppresses extracellular signal-regulated kinase (ERK) and mammalian target of rapamycin (mTOR) signaling associated with fibrosis and endoplasmic reticulum stress. Collectively, these actions preserve mitochondrial integrity, limit collagen deposition, and attenuate cardiac hypertrophy and fibrosis. Ub denotes ubiquitin. Blue arrows denote activation; red hammer lines indicate inhibition; black arrows represent stimulation; and double arrows signify restoration of impaired signaling.

Mitophagy plays a pivotal role in preserving mitochondrial quality under cardiac stress, and its dysregulation has been increasingly linked to pressure overload-induced hypertrophy and fibrosis. Impaired mitophagic flux contributes to mitochondrial dysfunction, endoplasmic reticulum (ER) stress, and inflammatory activation—factors that collectively exacerbate adverse cardiac remodeling [22]. In the present study, TAC induced autophagosome accumulation and disrupted mitochondrial architecture, consistent with mitophagy impairment. Cafestol treatment reversed these ultrastructural abnormalities and restored p62 expression, suggesting enhanced autophagic clearance and mitochondrial homeostasis. Mechanistically, cafestol may regulate mitophagy through its established effects on stress-responsive signaling pathways (Figure 7). Prior studies have shown that cafestol activates the Nrf2 pathway, which enhances antioxidant defenses and suppresses inflammasome activation and ER stress in both cardiac and hepatic models [6,23]. Nrf2 activation has also been reported to inhibit galectin-3 secretion and reduce fibrosis in pressure overload models [21]. Similarly, Qian Yang Yu Yin granules exert anti-inflammatory effects by inhibiting the NLRP3 inflammasome [24]. These findings support the hypothesis that cafestol’s modulation of mitophagy is part of a broader cytoprotective network involving oxidative stress reduction and immune regulation. Although earlier research has primarily emphasized the metabolic and antifibrotic properties of cafestol [8,25], its role in autophagy regulation is gaining recognition. In colon cancer models, cafestol activated LKB1/AMPK/ULK1-dependent autophagy, leading to tumor suppression [19]. While the cardiac context differs, the involvement of similar signaling intermediates suggests a conserved mechanism through which cafestol enhances autophagic flux. Moreover, cafestol’s ability to inhibit ERK phosphorylation—a pathway associated with autophagy suppression and fibrotic signaling—reinforces its potential to promote mitophagy [23,26]. Taken together, these findings highlight mitophagy as a promising therapeutic target in pressure overload-induced cardiac remodeling. Cafestol’s capacity to restore autophagic balance and preserve mitochondrial integrity complements its antifibrotic and antiapoptotic actions, offering a multifaceted foundation for mitochondrial quality-associated cardioprotection. Future studies should investigate the mechanistic interplay between cafestol and key mitophagy regulators such as the PINK1/Parkin and ULK1 pathways to fully elucidate its therapeutic profile.

### 4.2. Antifibrotic and Anti-Inflammatory Effects of Cafestol

Cardiac fibrosis and inflammation are defining features of pressure overload-induced remodeling, driven by fibroblast activation, immune cell infiltration, and extracellular matrix deposition. In this study, cafestol significantly reduced interstitial collagen accumulation and suppressed the expression of fibrotic markers, including CTGF, DDR2, α-SMA, and collagen I. These antifibrotic effects were accompanied by downregulation of inflammatory mediators such as CD68, CD44, and Gal3, indicating broad attenuation of both stromal and immune activation. Previous studies have shown that cafestol inhibits fibroblast proliferation and collagen synthesis in diabetic and high-glucose models, supporting its antifibrotic potential [8]. Its ability to suppress cyclic strain-induced inflammatory cytokines and adhesion molecules in endothelial cells further highlights its vascular anti-inflammatory activity [9]. In pressure overload models, Gal3 serves as a central mediator of fibrotic signaling and macrophage recruitment, and its inhibition by natural compounds such as fucoidan has been shown to alleviate cardiac remodeling [21]. The present findings suggest that cafestol exerts similar effects by modulating Gal3 and its downstream pathways. Mechanistically, cafestol’s inhibition of ERK phosphorylation may contribute to its antifibrotic efficacy, as ERK signaling promotes fibroblast activation and extracellular matrix deposition [23,26]. Additionally, activation of Nrf2, a transcription factor regulating antioxidant and anti-inflammatory responses, has been associated with reduced inflammasome activity and improved cardiac outcomes in hypertensive models [6,24]. These converging pathways govern oxidative stress and immune cell behavior, providing a mechanistic framework for cafestol’s dual antifibrotic and anti-inflammatory actions. Taken together, these results support the conclusion that cafestol mitigates pressure overload-induced cardiac remodeling by targeting key fibrotic and inflammatory mediators. Its modulation of Gal3, ERK, and Nrf2 pathways aligns with prior evidence and underscores its promise as a multi-targeted therapeutic candidate in cardiovascular disease, providing a basis for mitochondrial quality–associated cardioprotection.

### 4.3. Apoptosis and Endoplasmic Reticulum Stress Modulation

Apoptosis and ER stress are closely linked to mitochondrial dysfunction and represent key contributors to pressure overload-induced cardiac injury. In this study, TAC markedly increased the expression of proapoptotic markers Bax and cleaved caspase-3, along with ER stress indicators GRP78 and ATF4, reflecting maladaptive cellular stress responses. Cafestol treatment significantly reversed these alterations, suggesting a protective role in maintaining mitochondrial and ER homeostasis. The antiapoptotic effects of cafestol observed here are consistent with previous findings in ischemia–reperfusion and diabetic models, where it upregulated Bcl-2 and suppressed caspase activation [8,23]. Cafestol has also been shown to reduce cellular stress and promote survival under oxidative conditions by modulating ERK signaling, a pathway that governs both apoptosis and autophagy [23,26]. In hepatic and vascular systems, cafestol inhibits ERK-mediated inflammatory cascades, further supporting its role in stress attenuation [9,27]. Beyond ERK, cafestol activates the Nrf2 pathway, which enhances antioxidant defenses and alleviates ER stress by regulating molecular chaperones and unfolded protein response elements [6,24]. Nrf2 activation has been associated with reduced ATF4 expression and improved mitochondrial function, particularly in models of cardiotoxicity and hypertensive remodeling. These effects help preserve mitochondrial integrity and suppress apoptotic signaling, aligning with our ultrastructural findings of reduced mitochondrial disruption and autophagosome accumulation. Taken together, cafestol’s ability to modulate apoptosis and ER stress underscores its broader cytoprotective profile. By restoring the balance between pro- and anti-apoptotic signaling and alleviating ER stress, cafestol promotes cardiomyocyte survival under pressure overload. These mechanisms complement its mitophagy-enhancing and anti-inflammatory effects, reinforcing its therapeutic potential in cardiac remodeling and establishing a foundation for mitochondrial quality-associated cardioprotection.

### 4.4. Ultrastructural Insights from TEM

TEM enabled direct visualization of mitochondrial morphology and autophagic structures, providing critical insight into the subcellular effects of pressure overload and cafestol intervention. In TAC hearts, we observed pronounced mitochondrial swelling, cristae disruption, and autophagosome accumulation—hallmarks of impaired mitophagic clearance and organelle stress. These features are consistent with prior reports of mitochondrial vacuolar degeneration in hypertrophic and failing myocardium [28], reflecting a breakdown in mitochondrial quality control mechanisms. Cafestol treatment markedly preserved mitochondrial architecture, reduced autophagosome burden, and restored ultrastructural integrity. These findings align with evidence from other mitophagy-enhancing interventions, such as semaglutide and α-ketoglutarate, which improved mitochondrial morphology and suppressed inflammasome activation in pressure overload models [10,11]. Similarly, compounds such as fenofibrate and metformin have been shown to stabilize mitochondrial dynamics and prevent fragmentation through modulation of PINK1/Mfn2 and SIRT1 signaling [14,16], underscoring the therapeutic relevance of mitochondrial preservation. The observed reduction in autophagosome accumulation following cafestol treatment suggests enhanced mitophagic flux rather than autophagy inhibition—a distinction supported by studies demonstrating that mitophagy activation, rather than generalized autophagy suppression, is protective in pressure overload-induced cardiac remodeling [12,17]. Natural agents including Xinyang tablets and α-lipoic acid have also been reported to regulate the mitochondrial unfolded protein response and FUNDC1-mediated mitophagy, respectively, contributing to ultrastructural recovery and functional improvement [13,15]. Taken together, our TEM findings confirm that cafestol preserves mitochondrial integrity and restores autophagic balance at the ultrastructural level. These effects complement its molecular actions on p62 and apoptotic signaling, establishing mitophagy restoration as a central mechanism underlying its mitochondrial quality-associated cardioprotection. Future studies should incorporate expanded cohorts and quantitative TEM metrics, including morphometric scoring and autophagosome density analysis, to strengthen statistical power and validate ultrastructural outcomes with greater precision.

### 4.5. Dose-Dependent Effects and Translational Relevance

Graded administration of cafestol at 2, 10, and 50 mg/kg/day produced a dose-dependent attenuation of cardiac remodeling, with higher doses yielding more pronounced reductions in fibrosis, inflammation, and apoptotic signaling. This trend suggests a pharmacologically active range within which cafestol exerts its effects, likely through cumulative modulation of mitophagy, ER stress, and mitochondrial integrity. Comparable dose-dependent benefits have been reported for other mitophagy-enhancing agents, such as semaglutide and α-ketoglutarate, which improved cardiac function and inhibited NLRP3 inflammasome activation in pressure overload models [10,11]. Cafestol’s translational relevance is supported by its natural origin, oral bioavailability, and established dietary exposure. However, its metabolic effects, particularly on lipid profiles and hepatic function, warrant careful evaluation. Previous studies have shown that cafestol can alter serum cholesterol levels and hepatic enzyme activity, underscoring the need for targeted investigations into its long-term safety and metabolic impact. Future studies should incorporate lipid panel assessments, liver and kidney function tests, and chronic dosing protocols to assess tolerability and systemic effects. Pharmacokinetic profiling—including absorption, distribution, metabolism, and excretion parameters—will also be essential to define its therapeutic window and optimize dosing strategies. Bioactive compounds such as resveratrol and fenofibrate have similarly been shown to modulate mitochondrial dynamics and autophagic flux in a dose-dependent manner, improving cardiac outcomes in chronic disease models [14,18]. Moreover, interventions targeting the PINK1/Parkin and FUNDC1 pathways—such as metformin and α-lipoic acid—have demonstrated efficacy in restoring mitochondrial homeostasis and reducing cardiac injury, reinforcing the therapeutic potential of mitophagy-based strategies [13,16]. The ultrastructural and molecular improvements observed with higher doses of cafestol parallel those reported for pharmacological agents currently under clinical investigation. Its ability to engage conserved mitochondrial and autophagic pathways positions cafestol as a promising candidate for nutraceutical or adjunctive therapy in pressure overload-induced cardiac remodeling. Importantly, these findings raise the potential clinical implication that cafestol, as a modulator of mitochondrial quality control, may contribute to future strategies for cardiovascular disease management—pending further preclinical validation.

### 4.6. Limitations

While this study demonstrates promising cardioprotective effects of cafestol under pressure overload, several limitations should be acknowledged to contextualize its translational relevance. First, although TEM revealed improvements in mitochondrial morphology and autophagosome dynamics, we did not employ genetic or pharmacological tools to directly manipulate mitophagy-related pathways such as PINK1/Parkin, FUNDC1, or LC3-II. Prior studies using agents like berberine or α-lipoic acid have shown that targeted modulation of these axes can clarify causal relationships between mitophagy and cardiac outcomes [12,13]. Our findings therefore remain correlative, based on p62 accumulation and ultrastructural observations, and future work should incorporate mitophagy flux assays, pathway-specific inhibitors, or genetic models to validate whether cafestol directly modulates mitophagy or acts through upstream autophagic signaling. In addition, the absence of a positive control limits benchmarking of cafestol’s efficacy; comparative studies with established mitophagy activators such as α-ketoglutarate or berberine would help contextualize its effects. Second, the relatively short study duration of 28 days restricts evaluation of sustained therapeutic efficacy and safety. Extended follow-up is needed to assess long-term metabolic, hepatic, and vascular consequences of cafestol exposure, particularly given its known interactions with lipid metabolism and nuclear receptor signaling. Third, the study was conducted exclusively in male mice of a single strain and age range, consistent with prior TAC protocols, but this limits generalizability. Sex-specific differences in cardiac remodeling and mitochondrial stress responses have been reported [29]. underscoring the importance of including both sexes and diverse genetic backgrounds in future investigations. Finally, although the selected doses of cafestol (2, 10, and 50 mg/kg/day) produced dose-dependent therapeutic effects, pharmacokinetic parameters such as bioavailability, tissue distribution, and systemic exposure were not evaluated. These factors are critical for translating preclinical findings into clinical applications, as demonstrated in studies of metformin and fenofibrate, where mitochondrial targeting and exposure profiles were carefully characterized [14,16]. In summary, while this study highlights cafestol’s potential as a mitophagy-modulating agent in pressure overload–induced cardiac remodeling, further mechanistic validation, long-term pharmacological assessment, and sex-inclusive studies are essential to confirm its therapeutic applicability and safety profile.

## 5. Conclusions

This preclinical study demonstrates that cafestol attenuates pressure overload-induced cardiac remodeling through coordinated modulation of mitophagy, suppression of fibrotic and inflammatory signaling, and preservation of mitochondrial ultrastructure. Restoration of p62 expression, reduction in autophagosome accumulation, and normalization of mitochondrial morphology collectively support enhanced mitophagic flux and organelle homeostasis. These findings align with emerging evidence that targeting mitochondrial quality control—particularly via PINK1/Parkin, FUNDC1, and SIRT1 pathways—can mitigate cardiac hypertrophy and dysfunction [11,12,13]. While cafestol exhibited dose-dependent efficacy and favorable oral bioavailability in this murine model, its therapeutic potential remains exploratory. As with other mitophagy-enhancing compounds such as semaglutide and fenofibrate, further pharmacokinetic profiling, mechanistic validation, and long-term safety assessments are essential to define its therapeutic range and translational viability [10,14]. The integration of mitochondrial unfolded protein response and ER stress modulation, reported with Xinyang tablets and SOCS3-targeted interventions, may provide additional insight into cafestol’s multi-pathway regulatory effects [15,29]. Future investigations should examine molecular interactions between cafestol and key mitophagy regulators, including ULK1, Mfn2, and NAD^+^-dependent signaling pathways. Studies incorporating sex-inclusive designs, extended dosing regimens, and combination therapy with established cardioprotective agents will be critical to enhance clinical relevance. Moreover, given the growing interest in dietary strategies that support mitochondrial health, cafestol may serve as a prototype for nutraceutical-based approaches to cardiovascular disease prevention—pending further validation in translational models.

## Figures and Tables

**Table 1:** Echocardiographic measurements following cafestol administration in a murine model of TAC-induced cardiac hypertrophy. Parameters assessed include left ventricular end-diastolic volume (LVEDV), end-systolic volume (LVESV), stroke volume (SV), and diastolic mass (LVd Mass). Additional indices include left ventricular internal dimensions during systole (LVIDs) and diastole (LVIDd), posterior wall thickness at end-diastole (LVPWd), and interventricular septal thickness at systole (IVSs) and diastole (IVSd). Data are presented as mean ± SD (*n* = 5 per group). Statistical analysis was performed using one-way ANOVA with Tukey’s post hoc test. Normality and variance assumptions were verified using the Shapiro–Wilk and Levene’s tests. Effect sizes (*η*^2^) and 95% confidence intervals (CI) were calculated to aid interpretation. * *p* < 0.05, ** *p* < 0.01, *** *p* < 0.001 vs. sham controls; # *p* < 0.05, ## *p* < 0.01, ### *p* < 0.001 vs. TAC group.

	Sham	TAC	Cafestol-L	Cafestol-M	Cafestol-H
LVEDV (mL)	0.131 ± 0.036	0.208 ± 0.01 **	0.177 ± 0.021 #	0.195 ± 0.025	0.143 ± 0.025 ###
LVESV (mL)	0.038 ± 0.018	0.094 ± 0.021 **	0.058 ± 0.013 ##	0.068 ± 0.012 #	0.034 ± 0.012 ###
SV (mL)	0.093 ± 0.019	0.114 ± 0.018	0.119 ± 0.011	0.127 ± 0.028	0.109 ± 0.02
LVd Mass	0.112 ± 0.031	0.234 ± 0.034 ***	0.168 ± 0.013 ##	0.158 ± 0.023 ##	0.158 ± 0.007 ##
LVIDs	0.230 ± 0.043	0.332 ± 0.023 **	0.282 ± 0.02 ##	0.298 ± 0.017 #	0.232 ± 0.029 ###
LVIDd	0.370 ± 0.036	0.438 ± 0.007 **	0.414 ± 0.017 #	0.428 ± 0.019	0.384 ± 0.023 ##
LVPWd	0.084 ± 0.008	0.116 ± 0.008 ***	0.100 ± 0.000 ##	0.088 ± 0.01 ##	0.100 ± 0.000 ##
IVSs	0.112 ± 0.015	0.138 ± 0.012 *	0.138 ± 0.012	0.124 ± 0.017	0.144 ± 0.005
IVSd	0.084 ± 0.008	0.12 ± 0.0155 *	0.100 ± 0.000 #	0.096 ± 0.01 #	0.106 ± 0.008
EF	72.48 ± 6.049	54.82 ± 8.803 **	67.62 ± 4.214 #	64.46 ± 8.395	76.5 ± 7.052 ##

## Data Availability

The data presented in this study are available on reasonable request from the corresponding authors. The data are not publicly available due to privacy reasons.

## Supplementary Materials

The following supporting information can be downloaded at: https://www.mdpi.com/article/10.3390/nu17233680/s1, Figure S1: Crude immunoblotting data supporting mitophagy-related protein expression.

## Abbreviations

The following abbreviations are used in this manuscript:

TAC	Transverse Aortic Constriction
TEM	Transmission Electron Microscopy
HF	Heart Failure
ERK	Extracellular Signal-Regulated Kinase
mTOR	mammalian target of Rapamycin
BCA	Bicinchoninic Acid
PVDF	Polyvinylidene Fluoride
HRP	Horseradish Peroxidase
ECL	Enhanced Chemiluminescence
SD	Standard Deviation
ANOVA	Analysis of Variance
NIH	National Institutes of Health
IACUC	Institutional Animal Care and Use Committee
Gal-3	Galectin-3
Nrf2	Nuclear Factor Erythroid 2-Related Factor 2
SIRT1	Sirtuin 1
FUNDC1	FUN14 Domain Containing 1
LVEF	Left Ventricular Ejection Fraction
LVEDV	Left Ventricular End-Diastolic Volume
LVESV	Left Ventricular End-Systolic Volume
SV	Stroke Volume
LVIDs	Left Ventricular Internal Dimension at Systole
LVIDd	Left Ventricular Internal Dimension at Diastole
LVPWd	Left Ventricular Posterior Wall Thickness at Diastole
IVSs	Interventricular Septal Thickness at Systole
IVSd	Interventricular Septal Thickness at Diastole
