# Supplementary material for: Therapeutic Modulation of Mitophagy by Cafestol in Pressure Overload-Induced Cardiac Hypertrophy and Fibrosis
Source: Nutrients. 2025 Nov 25;17(23):3680. doi: 10.3390/nu17233680 (PMC12693951; doi:10.3390/nu17233680)
Supplement: Supplementary file 1 [file nutrients-17-03680-s001.zip › nutrients-3963263-supplementary.pdf]

GAPDH 36kDa ，n=3

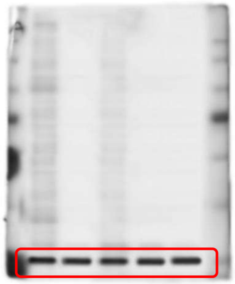

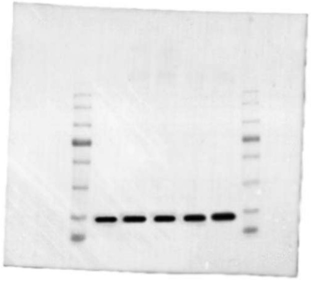

original image corresponding to the figure in the main article

CD44 82 kDa ，n=3

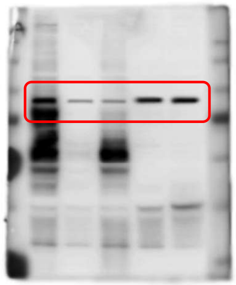

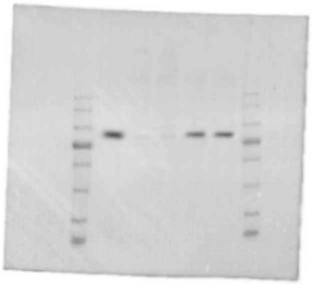

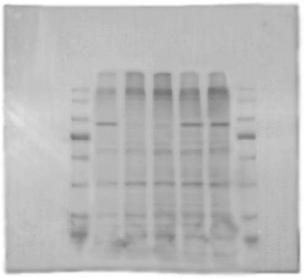

original image corresponding to the figure in the main article

CD68 110kDa ，n=3

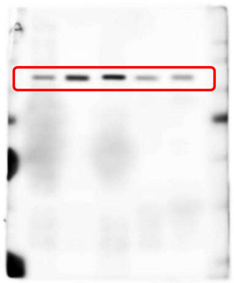

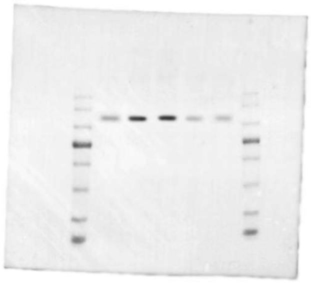

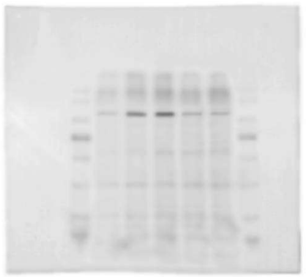

original image corresponding to the figure in the main article

collage Ⅰ 129 kDa ，n=3

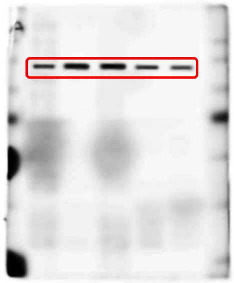

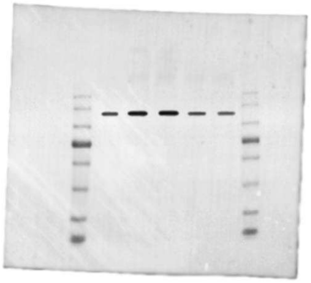

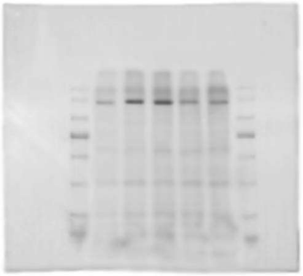

original image corresponding to the figure in the main article

CTGF 70 kDa ，n=3

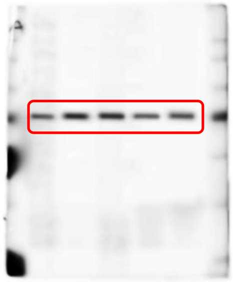

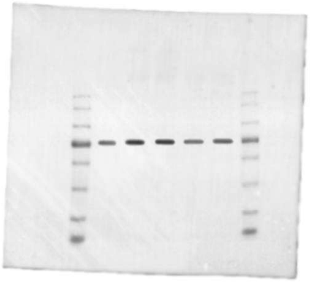

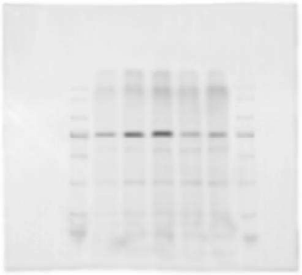

original image corresponding to the figure in the main article

DDR2 97 kDa ，n=3

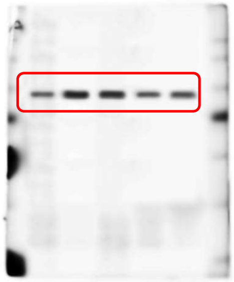

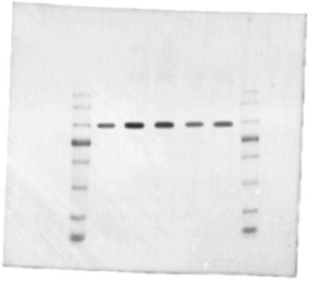

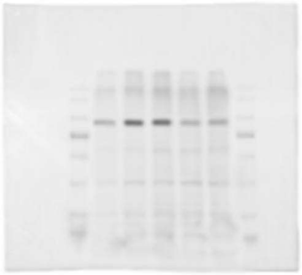

original image corresponding to the figure in the main article

Gal-3 28kDa ，n=3

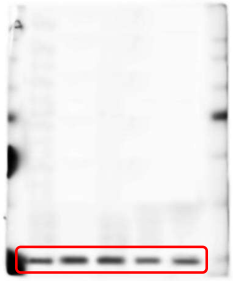

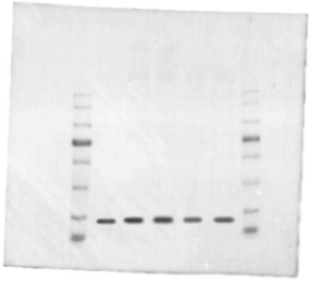

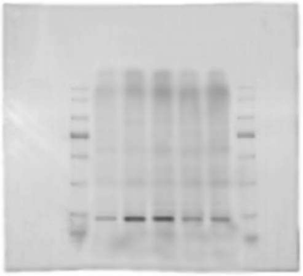

original image corresponding to the figure in the main article

α-SMA 68 kDa ，n=3

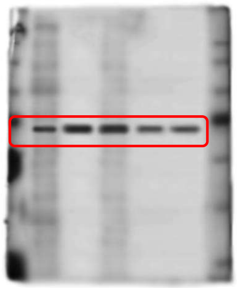

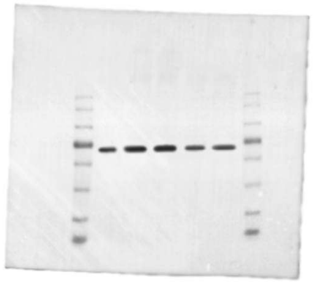

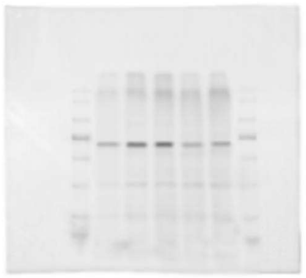

original image corresponding to the figure in the main article
